# Genome-guided isolation and characterization of a novel bacteriophage infecting *Escherichia coli* reveal a putative new genus

**DOI:** 10.3389/fmicb.2026.1909709

**Published:** 2026-08-20

**Authors:** Julia Oberdorfer, Jasmin Tesani, Thaysa Leite Tagliaferri, Simon Maurice Schmitz, Eva Miriam Buhl, Florian Kraft, Alex Krüttgen, Hans-Peter Horz

**Affiliations:** 1 Institute of Medical Microbiology, RWTH Aachen University Hospital, Aachen, Germany; 2 Electron Microscopy Facility, RWTH Aachen University Hospital, Aachen, Germany; 3 Center for Human Genetics and Genomic Medicine, Faculty of Medicine, RWTH Aachen University, Aachen, Germany; 4 Laboratory Diagnostic Center, RWTH Aachen University Hospital, Aachen, Germany

**Keywords:** *Escherichia coli*, lytic phage, new genus, outer membrane vesicles, *Vequintavirinae*

## Abstract

We have isolated and characterized a novel bacteriophage termed Jab, with lytic activity against multidrug-resistant clinical isolates of *Escherichia coli*. Phage Jab was identified from liquid manure by means of metagenome sequencing of a phage community enrichment using an *E. coli* clinical isolate ECH07 as host. The initial enrichment was composed of four phages, of which phage Jab represented only a minute fraction (less than 1%). Jab isolation strategy comprised a targeted approach using iterative replication rounds while equipping ECH07 with resistance against the numerically dominant phages coupled with a subsequent host switch to *E. coli* BL21. Whole-genome sequence analysis revealed only a remote evolutionary distance to known phages within the subfamily *Vequintavirinae*. The dsDNA genome of phage Jab comprises 142,100 bp (GC content 40.09%) and encodes 264 proteins and five transfer RNAs (tRNAs). No lysogeny-associated proteins were detected, suggesting an obligate lytic lifestyle. *In silico* genome analysis revealed the presence of at least four putative depolymerases. The closest homology of phage Jab is with members of the new genus *Septuagintavirus* with around 34% nucleotide identity. VIRIDIC and network analyses strongly suggest that phage Jab belongs to a putative novel genus. The host range of phage Jab is likely restricted to *E. coli*, displaying a moderately narrow host range (i.e., productive lysis in 8 out of 27 isolates tested). Notably, transmission electron microscopy (TEM) revealed the occurrence of conspicuous unique spherical structures attached at the end of the tail fibers when propagated on BL21 but not when propagated on ECH07. Although their function remains enigmatic, the possible role of those structures as a bacterial (vesicle-based) defense mechanism warrants further investigation.

## 1 Introduction

Our understanding of viral diversity and virus-host interactions has greatly improved with the advent of culture-independent technological advances such as high-throughput viral sequencing and metagenomic analysis of large viral communities (Roux and Coclet, 2026; Paez-Espino et al., 2016). With that, genomes of uncultivated viruses can be reconstructed *de novo* from biotic and abiotic environments without laboratory isolation of the virus-host system. Reconstructed viruses are then classified as novel through their sequence similarity to known viruses, increasing the number of diverse non-cultured viruses to millions in the last decade alone (Roux et al., 2019).

However, culture-dependent methods remain critical in advancing our understanding of biological systems. They allow characterization of bacteriophages (phages for short) and provide crucial information about virus-host interactions, viral life cycles, infectivity, and ecological roles (Dion et al., 2020). Moreover, in light of the antibiotic crisis, ongoing phage isolation and characterization for phage therapy are gaining more attention (Unterer et al., 2023). In contrast, the diversity of phages known and currently used for antibacterial applications falls far short of the true phage diversity found in nature (Pye et al., 2024).

Unlike advancements in metagenomic techniques, culture-based methods employed for phage isolation have remained largely unchanged since the very first steps of phage research, possibly leading to bias in phage isolation and reducing diversity (d'Herelle, 1926). Following phage enrichments from environmental samples, phage isolation is typically performed with the double-layer plaque assay and the selection of individual plaques. With this method, plaque morphology and -size serve as key indicators for distinct phage populations (Hyman, 2019). Additionally, phage isolation can also be biased toward phages with a significantly higher abundance in the environmental starting material and/or toward phages that propagate more efficiently under the laboratory enrichment conditions (Zhang et al., 2026).

Here, we report the novel phage Jab, able to infect the multidrug-resistant clinical *Escherichia coli* isolate ECH07. This isolate harbors the OXA-48 oxacillinase, one of the most frequent carbapenemases of clinical relevance (Peirano and Pitout, 2025). To this end, phage enrichment was analyzed using metagenomic sequencing to capture the full diversity of phages that were able to propagate on ECH07. Within this phage consortium, a unique phage (termed Jab) was identified. Due to its low abundance within this phage enrichment, an alternative approach using targeted isolation was performed by induction of resistance against competing phages combined with host switching. Although various phages against *E. coli* are already described in the literature and databases, phage Jab shows only a few genomic similarities to known phages and likely represents a member of a novel phage genus.

## 2 Materials and methods

### 2.1 Host bacteria and culture conditions

A total of 18 multidrug-resistant, OXA-48-positive clinical isolates of *E. coli*, obtained from urinary tract infection, groin swab, anorectal swab, blood culture, tissue sample, and wound infection, were collected from various clinical specimens at the RWTH Aachen University Hospital, Germany. All samplings were performed in compliance with relevant laws and institutional guidelines and in accordance with the ethical standards of the Declaration of Helsinki. All isolates were cultured in lysogeny broth (LB) (NaCl 1% w/v, tryptone 1% w/v, yeast extract 0.5% w/v) and stored at -80 °C. In addition, nine *E. coli* reference strains were included in this study. Species identity of all isolates was verified via Matrix-Assisted Laser Desorption/Ionization-Time of Flight (MALDI-TOF) mass spectrometry (Microflex LT, Bruker Daltonik GmbH, Bremen). Antimicrobial susceptibility testing was performed using the VITEK2 system (bioMérieux, Marcy-l'Étoile, France). The identification of carbapenem-resistance genes was verified by specific polymerase chain reactions (PCR) using the Xpert Carba-R kit (Cepheid, Inc., USA). Resistance profiles are shown in Supplementary Figure S1. Before use in experimental settings, bacterial strains were generally grown beforehand in LB at 37 °C, 200 rpm overnight.

### 2.2 Phage enrichment from slurry

A slurry sample collected from an organic farm near Roetgen municipality (50° 38' 54.64" N, 6° 11' 46.25" E), North Rhine-Westphalia, Germany, was used as a source of novel phages. Slurry filtrates were prepared by homogenizing 10 g of slurry in 40 ml LB medium using a magnetic stirrer for 30 min. 5 ml of bacterial cultures were added and incubated at 37 °C overnight. The tubes were centrifuged at 4,000*g* in a swing bucket rotor for 10 min. The resulting supernatants were transferred to new tubes, and centrifugation was repeated once again at 10,000*g*. The supernatants were subsequently filtered through a 0.45 μm pore syringe-mounted polyethersulfone (PES) membrane filter. Finally, 5 ml of the filtrate was again incubated with 5 ml of LB medium and 200 μl of host bacterial strain at 37 °C overnight at 200 rpm. The resulting phage enrichment was again subjected to centrifugation followed by sterile filtration of the supernatant.

### 2.3 Bacteriophage DNA extraction

Bacterial DNA was removed by a DNase digestion for 15 min at 37 °C with subsequent inactivation of the DNase for 10 min at 75 °C. This was followed by treatment with Proteinase K (20 μl) for 30 min at 56 °C. For the metagenome sequencing approach of the initial phage enrichment (Illumina platform), phage DNA isolation was performed with the QIAamp DNA Mini Kit (Qiagen, Hilden, Germany). For Nanopore sequencing of the isolated phage Jab, phage DNA extraction was performed with phenol-chloroform. This was done by extracting the phage lysates twice with an equal volume of phenol/chloroform/isoamyl alcohol 25:24:1. The DNA solution was then washed once with chloroform and precipitated overnight at -20 °C with 2 volumes of isopropanol. The DNA was pelleted, washed twice in 70% ethanol and suspended in elution buffer.

### 2.4 Whole genome sequencing

#### 2.4.1 Illumina-based metagenome sequencing of enriched phage community and genome assembly

DNA was subjected to random shotgun sequencing, using the Nextera XT DNA Library Preparation Kit (Nextera Flex, Illumina DNA Prep, (M) Tagmentation) on an Illumina HiSeq 2500 platform at NGS Competence Center Tübingen. Raw Illumina paired-end reads were quality-checked using FastQC and assembled using metaSPAdes by Nurk et al. (2017) with standard parameters. The quality and completeness of the contig as well as the terminal repeats were evaluated using CheckV version 1.0.1 with standard settings (Nayfach et al., 2021).

#### 2.4.2 Nanopore sequencing of isolated phage Jab and genome assembly

To confirm the genome sequence of phage Jab and to investigate the structure of bacteriophage genome termini and packaging mode, an additional sequencing effort was made using the Oxford Nanopore Technologies platform on the plaque-purified bacteriophage DNA. Briefly, phage Jab was propagated on 20 ml broth culture of *E. coli* ECH07 as described above. The phage particles were concentrated from the filtrate by ultrafiltration using Amicon Ultra centrifugal filters, and the DNA was extracted using phenol-chloroform. The DNA was sheared using the Megaruptor 2 device (Diagenode S.A., 4102 Seraing, Belgium) and cleaned up with 0.4 × CleanNA CleanNGS beads (CleanNA B.V., Waddinxveen, The Netherlands). Nanopore sequencing was carried out according to the manufacturer's protocol, using the EXP-NBD104 together with the SQK-LSK109 on a FLO-FLG001 flow cell (Oxford Nanopore Technologies plc, Oxford, UK). Base calling was carried out with Guppy version 6.4.2. The resulting FASTQ files were used for assembly with Flye version 2.9.3. The Flye assembly was polished using two rounds of Racon version 1.4.3 and two rounds of Medaka version 1.11.2. Finally, short-read data was used to polish the long-read assemblies with five rounds of pilon version 1.24.

### 2.5 Targeted isolation of phage Jab from other phages

Primers were designed against all phages identified in the initial enrichment, based on the metagenomic sequencing data. This included the unique phage Jab as well as three additional phages, referred to as Twora, Threena, and Foura. Real-time quantitative PCR (qPCR) was performed from DNA extracts of the initial phage enrichment and from washed double-layer plaque assay (DLPA) plates to determine the relative proportions of each of the four phages. Prior to DNA extraction, samples were treated with DNase I. The most abundant phage Foura was purified by three consecutive single plaque isolations and propagations using the DLPA as follows. The sample was diluted with LB medium by 10-fold dilution. The diluted sample (100 μl), the host bacteria culture (100 μl), and LB top agar containing 0.35% agar (3 ml) were mixed and poured onto the LB agar plate containing 1.5% agar, followed by incubation at 37 °C for 24 h. Single plaques were obtained by touching them with a needle and resuspending them in 5 ml LB medium and 100 μl bacteria in log phase for propagation.

Resistance to the most abundant phage Foura was generated in *E. coli* strain ECH07 by incubation of phages and bacteria in liquid culture. After 16 h of incubation, the bacteria were streaked onto LB agar plates followed by picking single colonies after overnight incubation. Phage resistance was assumed if no lysis occurred in the spot testing assay on plated colonies.

The Foura-resistant ECH07 was used as a new host for continued propagation of the initial phage enrichment containing all four phages, but to prevent multiplication of the abundant phage Foura. Resistance to the subsequently emerging dominant phage Twora was induced as described above. Afterwards, the propagation in *E. coli* strain BL21 was repeated with the resulting lysate four times, and finally phage Jab could be purified by three consecutive single plaque isolations and propagations. qPCR was performed with designed primers to verify the identity of the phage.

The PCR primers Jab_F (5′-AGCGGGTCTTGTAGGGTTTTAAC-3′) and Jab_R (5′-GCAATGCACCACTAAGGGC-3′), as well as Jab2_F (5′-GTGCGTGGCTCTATTTCTG-3′) and Jab2_R (5′-CAAGACCCGCAGAAACAACA-3′) were developed for detection and quantification of phage Jab in enrichments based on either *E. coli* BL21 or ECH07 as host. The primers Twora_F (5′-GCAGCGGTACAGTAAAGAGC-3′) and Twora_R (5′-GAGGGTAGGCAATGCAATGG-3′), Threena_F (5′-TGGGCTTGTTCTACGCTGTATTG-3′) and Threena_R (5′-TGAGCGTCAACCATACACC-3′), Foura_F (5′-TGGCTCCTGATGTTCGTC-3′) and Foura_R (5′-GCTGACGGGTGGAATGATTC-3′) were developed for quantification of phages Twora, Threena and Foura. Amplification and detection of phage DNA by qPCR was performed using the SYBR Green Master Mix (Bio-Rad, United States). The thermal profile of the PCR began with an initial denaturation step at 95 °C (10 min), followed by 39 cycles of denaturation at 95 °C (30 s), primer annealing at 58 °C (30 s) and extension at 72 °C (1 min) as well as a further extension cycle at 72 °C (5 min). All samples were run in triplicate, including the negative control. PCR efficiency was assessed by running qPCR of the four PCR assays (each specific for one phage) on 10-fold serial dilutions of the test samples. Efficiency was calculated from the slope of the resulting standard curves. In the next step, the crossing points (CPs) observed for each of the four qPCRs were used for pairwise comparisons of the fold differences between phage Jab and the other phages after correcting for the different PCR efficiencies. The fold differences could then be transferred into relative abundances (percentage values) of the four phages in the enrichments. This quantitative approach provides only approximate values. This is due to the absence of external standards with defined DNA concentrations, which would have enabled more accurate determination of PCR efficiencies and calculation of the absolute number of phage genomes. However, for our purpose of targeted phage isolation, the changes in CP values after each round of phage enrichment provided practical and sufficient information to assess shifts in the relative abundance of phage Jab. The resulting DNA fragments were then run on a 1.5% Tris-Borate-ethylenediaminetetraacetic acid (EDTA) (TBE) agarose gel at 80 V for 3 h and visualized using the GelStudio SA system (Analytik Jena, Jena). Sanger sequencing was performed to verify the reaction specificity of the different PCR assays.

### 2.6 Plaque purification and phage propagation

The isolated phages were propagated in *E. coli* strain BL21. The sample was purified from bacteria by centrifugation (Eppendorf 5810R, Hamburg, Germany) at 10,000*g* for 10 min followed by sterile filtration of the supernatant using a 0.45 μm-pore-size and a 0.22 μm-pore-size sterile filter (Filtropur S, Sarstedt, Nürnbrecht, Germany).

### 2.7 Genome sequence analysis

Phage GC content was calculated from the final assembled genome sequence using GeneMarkS (Besemer et al., 2001) as part of the genome annotation pipeline. Gene identification was done following Salisbury and Tsourkas (2019). Functional annotation of identified genes was performed using Prokka (Seemann, 2014) with Basic Local Alignment Search Tool for proteins (BLASTp) searches against the NCBI nr database and Hidden Markov Model (HMM) searches against the Prokaryotic Virus Remote Homologous Groups (PHROGS) database. The circular map of Jab was visualized using CGView (Stothard et al., 2019). The phage lifestyle was predicted by BACPHLIP (Galaxy Version 1.0) (Hockenberry and Wilke, 2021), which predicts lifestyle based on conserved domains of integrases, excisionases, recombinases, transposases, and partitioning proteins (ParA and ParB). The presence of genes encoding putative depolymerases (DPOs) was assessed *in silico* using the DPO prediction models DePP and PhageDPO (Magill and Skvortsov, 2023; Vieira et al., 2025).

#### 2.7.1 Network analysis

A total of 28,665 phage genomes (2,484,471 proteins), almost complete phage genomes from GenBank from April 2025, were downloaded via inphared.pl (INfrastructure for a PHAge REference Database; Cook et al., 2021) and combined with Escherichia phage Jab for calculating a gene-sharing network using vContact2 (Bin Jang et al., 2019). The protein-sharing network of a subset of 225 phage genomes (including phage Jab), chosen based on their similarity scores with Escherichia phage Jab, was visualized using Cytoscape version 3.10.3 (Kohl et al., 2011). The prefuse force-directed layout was selected with the shared protein cluster-based similarity scores as the weight [based on the Markov Cluster Algorithm (MCL clustering method)], arranging viral genomes that share more viral protein clusters (PCs) closer together. Edges were treated as unidirectional in the network.

### 2.8 Phylogenetic analysis and taxonomic assignment

The genome-based taxonomy was conducted according to the guidelines of Turner et al. (2021). Nucleotide identity was calculated by multiplying percent identity and percent coverage of the BLASTn search. To understand the phylogenetic relationships between phages, complete nucleotide sequences of all phages from Viral Cluster 981 as well as the 30 closest relatives identified by a BLASTn search were submitted to the Virus Classification and Tree Building Online Resource (VICTOR) web service (https://victor.dsmz.de), a method for the genome-based phylogeny and classification of prokaryotic viruses (Meier-Kolthoff and Göker, 2017). Duplicate and non-associated sequences were removed. All pairwise comparisons of nucleotide sequences were performed using the Genome-BLAST Distance Phylogeny (GBDP) method by Meier-Kolthoff et al. (2013) under the settings recommended for prokaryotic viruses (Meier-Kolthoff and Göker, 2017). The resulting intergenomic distances were used to infer a balanced minimum evolution tree with branch support via FASTME, including Surface Plasmon Resonance (SPR) postprocessing (Lefort et al., 2015) for each of the formulas D0, D4, and D6, respectively. Branch support was inferred from 100 pseudo-bootstrap replicates in each case.

Taxon boundaries at the species, genus, and family level were estimated with the OPTSIL program (Göker et al., 2009), the recommended clustering thresholds (Meier-Kolthoff and Göker, 2017), and an *F*-value (fraction of links required for cluster fusion) of 0.5 (Meier-Kolthoff et al., 2014). The phylogenomic GBDP trees were inferred using the D0, D4, and D6 formulas, yielding average support of 18, 21, and 22%, respectively. Accordingly, the tree with the D6 formula providing maximum average support was recruited in the analysis. Additionally, VIRIDIC was used with default settings to calculate the intergenomic similarities of phage Jab with its closest relatives (Moraru et al., 2020).

Moreover, amino acid sequences of the terminase large subunit (TerL) and major head protein were used for marker gene phylogenies. The homologs were retrieved from the corresponding viral PCs built by vConTACT2. Short sequences < 101 amino acids and sequences that did not show common sites with phage Jab in the alignment were excluded from the study. The neighbor-joining phylogenetic trees were generated using MEGA 7 with partial deletion treatment at standard settings after the multiple sequence alignments using ClustalW (Kumar et al., 2016). The resulting tree was rooted at the midpoint with branch support inferred from 10,000 pseudo-bootstrap replicates. Genomic comparisons between phages were conducted with tBLASTx version 2.16.0 and visualized by running Easyfig version 3.0.0 (Sullivan et al., 2011) as a Python script.

### 2.9 Nucleotide sequence accession number

The genome nucleotide sequence of phage Jab has been deposited in GenBank under accession number PZ476263, following Roux et al. (2019).

### 2.10 Transmission electron microscopy imaging

For transmmission electron microscopy (TEM), samples were prepared by transferring the phage lysate to 4-(2-hydroxyethyl)-1-piperazineethanesulfonic acid (HEPES) buffer via Amicon Ultra Centrifugal Filter Units 100K (Merck Darmstadt, Germany). The samples were allowed to adsorb onto glow-discharged formvar-carbon-coated nickel grids (Maxtaform, 200 mesh, Plano, Wetzlar, Germany) for 10 min. The grids were washed three times with aqua bidest. Negative stain was performed with 1% neodymium acetate (Sigma-Aldrich, St. Louis, USA). The samples were examined using a Hitachi HT7800 transmission electron microscope (Hitachi, Tokyo, Japan) operating at an acceleration voltage of 100 kV. Characterization of phage morphology and determination of phage head and tail size was carried out using ImageJ (Schneider et al., 2012) by analyzing multiple phages (*n* = 10).

### 2.11 Determination of host range and efficiency of plating

The host range of the phage was evaluated using the spot testing assay. Briefly, 15 μl of purified phage lysate, diluted in 10-fold steps, was spotted onto bacterial lawns, with 400 μl of each test strain plated onto LB agar plates, followed by overnight incubation at 37 °C. Phage plaques were manually inspected to distinguish productive infection (lysis with individual phage plaques formed) and lysis from without (lysis without individual phage plaques). Efficiency of plating (EOP) was calculated by dividing the phage titer at the terminal dilution on the challenge strain by the titer of the same phage on the bacterial strain that yielded the highest phage titer.

### 2.12 Outer membrane vesicle isolation

Outer membrane vesicle (OMV) isolation of *E. coli* strains BL21 and ECH07 was performed as previously described (Reyes-Robles et al., 2018). *E. coli* cells were struck out onto an LB agar plate and incubated overnight at 37 °C. The following day, an individual colony was inoculated into 6 ml LB broth and grown for 8 h at 37 °C. This culture was then subcultured in a 1:100 ratio in 600 ml LB broth and incubated overnight at 37 °C. The cultures were chilled in an ice-water bath for 10 min and then centrifuged for 10 min at 10,000*g* to pellet the bacterial cells. The supernatants containing the outer membrane vesicles were filtered through a SteriCup 0.22 μm filter system (Millipore) to remove any remaining bacterial cells, and an EDTA-free protease inhibitor cocktail was added. The outer membrane vesicles were pelleted from the culture filtrates by centrifugation at 28,000 rpm for 2 h and 15 min. After removal of the supernatants by decanting, the outer membrane vesicle pellets originating from a 600 ml culture were resuspended in a total of 500 μl phosphate-buffered saline solution and stored in aliquots at -80 °C. The interactions between OMVs and phage Jab were studied using TEM imaging. 100 μl of phage lysate at a concentration of 10^7 pfu/ml were mixed with 10 μl OMVs in PBS and incubated for 1 h at 37 °C. This mixture was then transferred to HEPES buffer via Amicon Ultra Centrifugal Filter Units 100K (Merck Darmstadt, Germany), and TEM was performed as described above.

## 3 Results

### 3.1 Composition of phage enrichments and successive isolation of phage Jab

Phage enrichment was based on the clinical *E. coli* isolate ECH07 incubated overnight in a slurry sample. Metagenome sequence analysis of this enrichment revealed the presence of four distinct phages, referred to as phage Jab, Twora, Threena, and Foura. While phage Jab showed only a remote genomic relationship to known phages, the other three phages were closely related to already known phages. The numerical abundance of Jab was very low, as deduced from the relative proportions of sequence reads matching its genome.

The BLASTn search results against sequences in the GenBank NCBI databases were as follows: Phage Twora, a putative member of the genus *Tequintavirus*, exhibited nucleotide identities of 83.25, 78.72, and 78.19% with its closest relatives Salmonella phage VSe12 (NC_048794), Escherichia phage vB_EcoS_ESCO40 (OM386660) and Escherichia phage Lucris (PV806338), respectively. Phage Threena, a putative member of the genus *Asteriusvirus*, shared 88.67, 76.95, and 75.74% with its closest relatives Escherichia phage A4 (ON286972), Escherichia phage vB_Ec-M-J (OQ718158) and Escherichia phage Turkey3 (PQ471515), respectively. Phage Foura, a putative member of the genus *Suseptimavirus* shared nucleotide identities of 92.15, 85.71, and 85.71% with Escherichia phage vB_EcoP_SU7 (NC_070980), Escherichia phage vB_EcoP-101114UKE3 (NC_070982.1) and Escherichia phage vB_EcoP-101114BS3 (MZ234015), respectively. In contrast, BLASTn searches of phage Jab showed remarkable low nucleotide identity with the closest phylogenetic relation to members of the recently proposed genus *Septuagintavirus*, that is, Escherichia phage A7_1 (OP795442), Escherichia phage A5-4 (OP744025) and the unclassified Escherichia phage vB_EcoM-LTH01 (PP430345), sharing only 34.50, 34.46, and 34.59% nucleotide identity, respectively.

**Figure 1:**
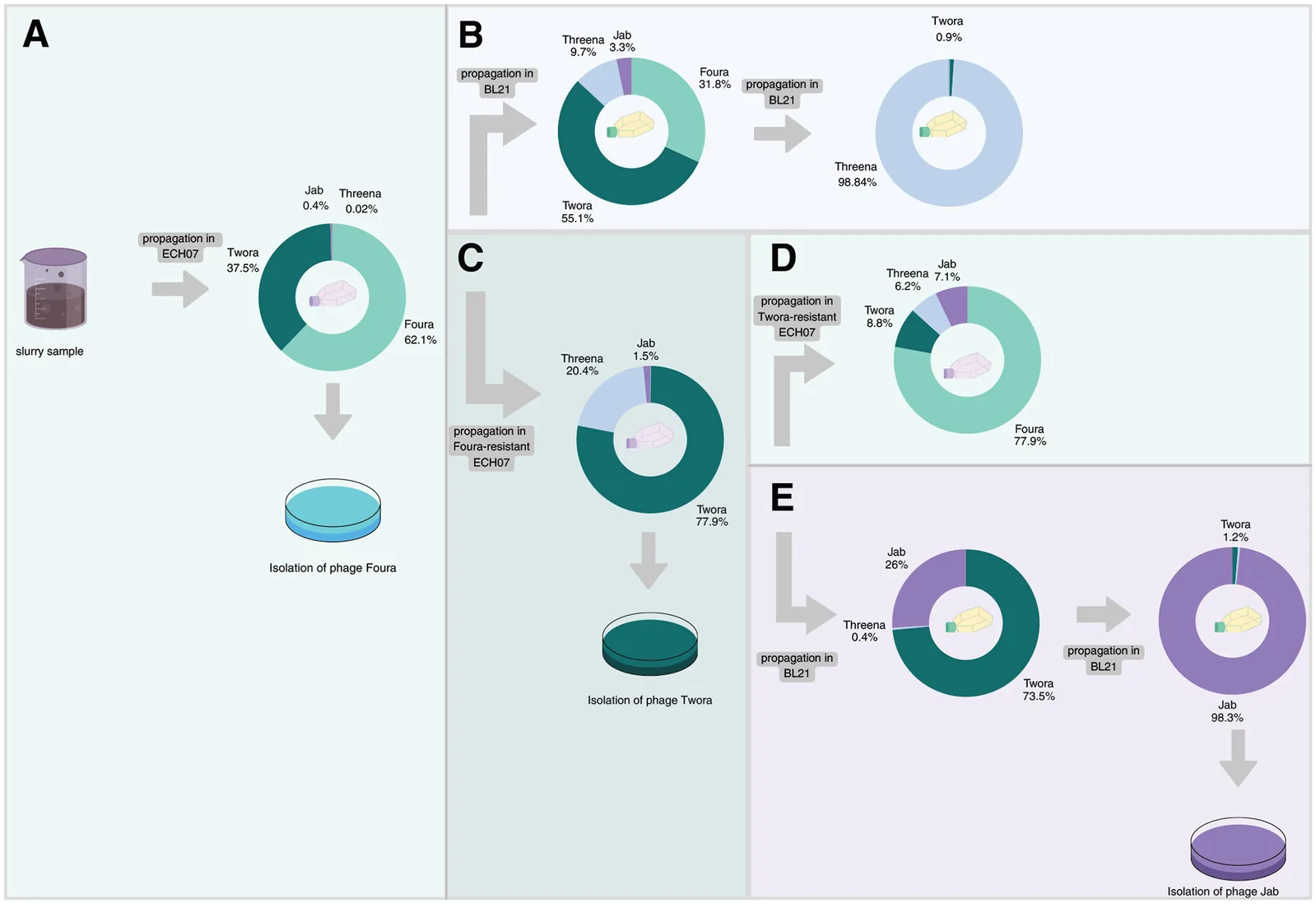
Schematic representation of the isolation strategy for phage Jab. **(A)** Initial phage enrichment based on a slurry sample and the *E. coli* host ECH07. As a result, four different phages (named Jab, Twora, Threena, and Foura) had been enriched at different proportions. **(B)** Follow-up enrichments using *E. coli* BL21 as host. **(C)** Follow-up enrichment using ECH07 with resistance against phage Foura. **(D)** Follow-up enrichment using ECH07 with resistance against phage Twora. **(E)** Follow-up enrichments using *E. coli* BL21 as host. The proportions depicted are approximate values based on crossing points (CPs) derived from qPCR. Note that qPCR-based quantification reflects genome copy number (potentially also including non-infectious particles). Therefore, the indicated values may not strictly reflect the proportion of infectious phage particles. Due to the small magnitude of certain phage proportions, not all values are labeled in the pie chart.

To further investigate the biological properties and confirm the genome sequence of the phage Jab, a targeted isolation was carried out. First, phage-specific qPCR analysis confirmed the low abundance of phage Jab in the enrichment with only 0.4%, as opposed to the abundance of phage Twora and Foura with 37.5 and 64.1%, respectively (Figure 1A). Accordingly, direct attempts to isolate phage Jab using the double-layer plaque assay failed, while it was easy to isolate phage Foura from its numerically dominating plaques on solid medium (Figure 1A). To enhance the propagation of phage Jab, we pursued two different enrichment strategies. First, by host switching using *E. coli* BL21, a consecutive enrichment increased the relative proportion of Jab to 3.3%. However, another successive enrichment step again using *E. coli* BL21 as host exclusively boosted phage Threena propagation, while phage Jab went extinct (Figure 1B). The other strategy relied on suppressing the overabundant phage Foura by an enrichment step using a previously generated Foura-resistant ECH07 strain as host. As a result, the relative proportion of phage Jab slightly increased; however, this enrichment step particularly fostered propagation of phage Twora, making it now about 78% of the phage mixture (Figure 1C). Subsequent isolation of phage Twora enabled the generation of a Twora-resistant ECH07 strain, which was already resistant to phage Foura. However, the next enrichment based on the phage Twora-resistant bacterial strain ECH07 resulted in a strong recurrence of phage Foura (Figure 1D). Therefore, we decided to use the same enrichment mixture for a successive round of enrichment by again switching the bacterial host to *E. coli* strain BL21. As a result, phage Jab made up 26% of the resulting phage mixture (Figure 1E). Another round of enrichment with BL21 then increased the relative proportion of phage Jab to 98.3%, after which its isolation and purification using the double-layer plaque assay was successful (Figure 1E).

### 3.2 Genomic characteristics

#### 3.2.1 Genome analysis

**Figure 2:**
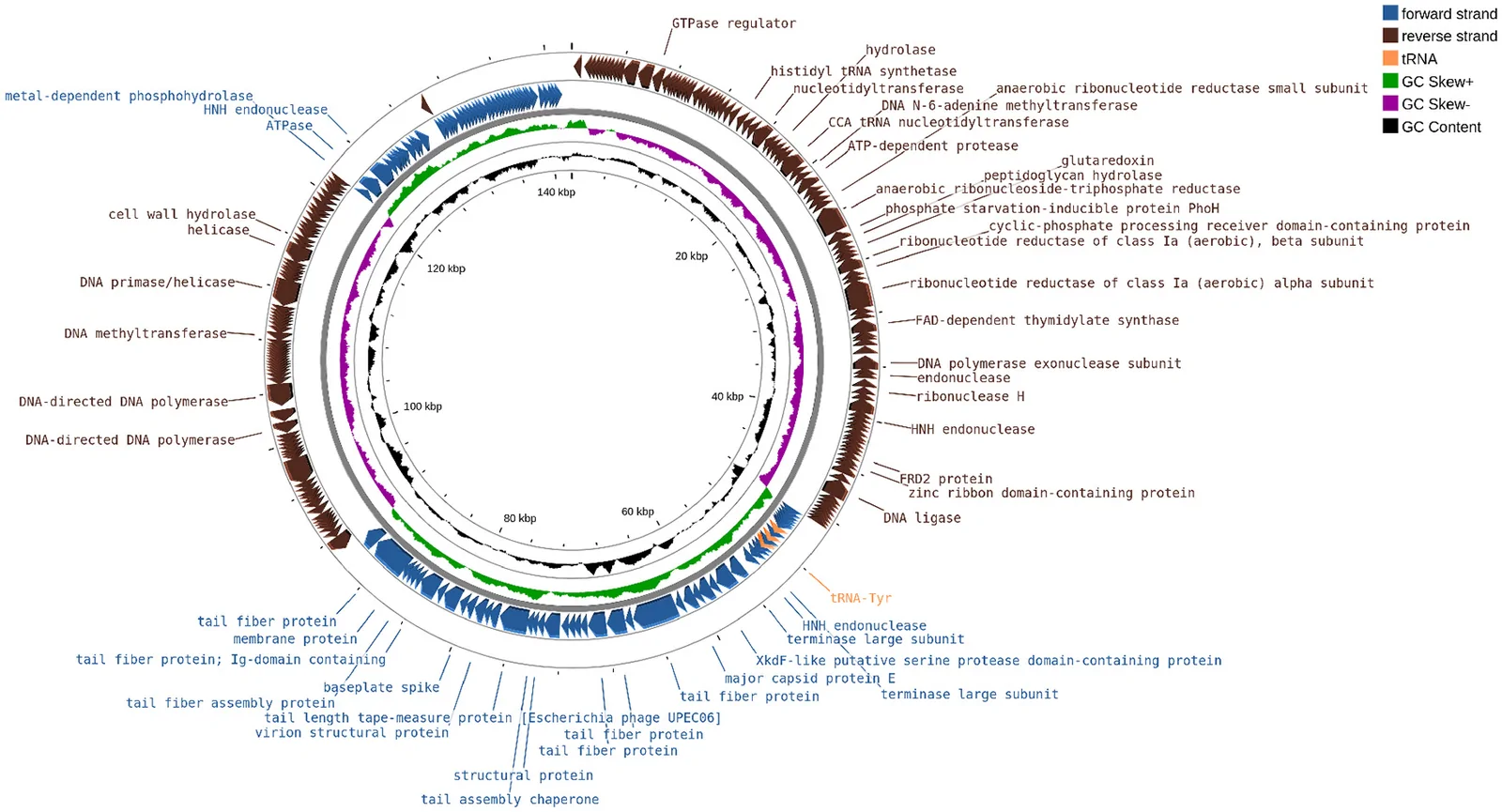
Circular genome map of phage Jab. The GC content (black) is shown in the innermost circle, followed by the GC skew (green and purple); the forward strand with protein-coding genes (blue); the reverse strand with protein-coding genes (brown), shown in the outermost circle; tRNA genes are found only in the forward strand (orange).

Phage Jab has a relatively large dsDNA genome comprising 142,100 bp with a GC content of 40.09%. The completeness of the genome could be verified by CheckV, which also identified direct terminal repeats of 475 bp in length. A total of 264 protein-coding genes (171 on the negative strand and 93 on the positive strand) plus five tRNA genes (encoding tRNA-Met, tRNA-Tyr, tRNA-Gly, tRNA-Gln, and tRNA-Pro) were predicted in the genome of phage Jab. The majority (83%), of the 264 predicted protein coding genes encode hypothetical proteins without a known function, and 46 proteins (17%) were annotated with putative functions, that is, related to virion assembly (6%), DNA-related processes (7%) host lysis (1%), and various other processes (3%; Figure 2 and Supplementary Table S1). According to the annotation results, the genome does not contain Open Reading Frames (ORFs) putatively encoding integrases, excisionases, or repressor proteins. This means that phage Jab likely represents an obligate lytic virus. The absence of lysogeny-associated proteins could furthermore be verified based on BACPHLIP (Hockenberry and Wilke, 2021). Notably, for 32 ORFs (12%), no hits were found using BLASTn searches (Supplementary Table S1). *In silico* analysis revealed the presence of at least four putative depolymerases encoded in the genome of phage Jab, with prediction probabilities ranging from 71 to 96%, that is, ORF 13, 51, 140, and 146 (Supplementary Table S2). In addition, 17 hypothetical proteins according to the annotation via BLASTn and Interpro could be categorized as structural proteins according to their relatively close genetic homology to structural proteins of phage W70, which were identified via electrospray-tandem mass spectrometry (Cortés-Martín et al., 2024; Supplementary Table S1).

#### 3.2.2 Phylogenetic and comparative genomic analysis

**Figure 3:**
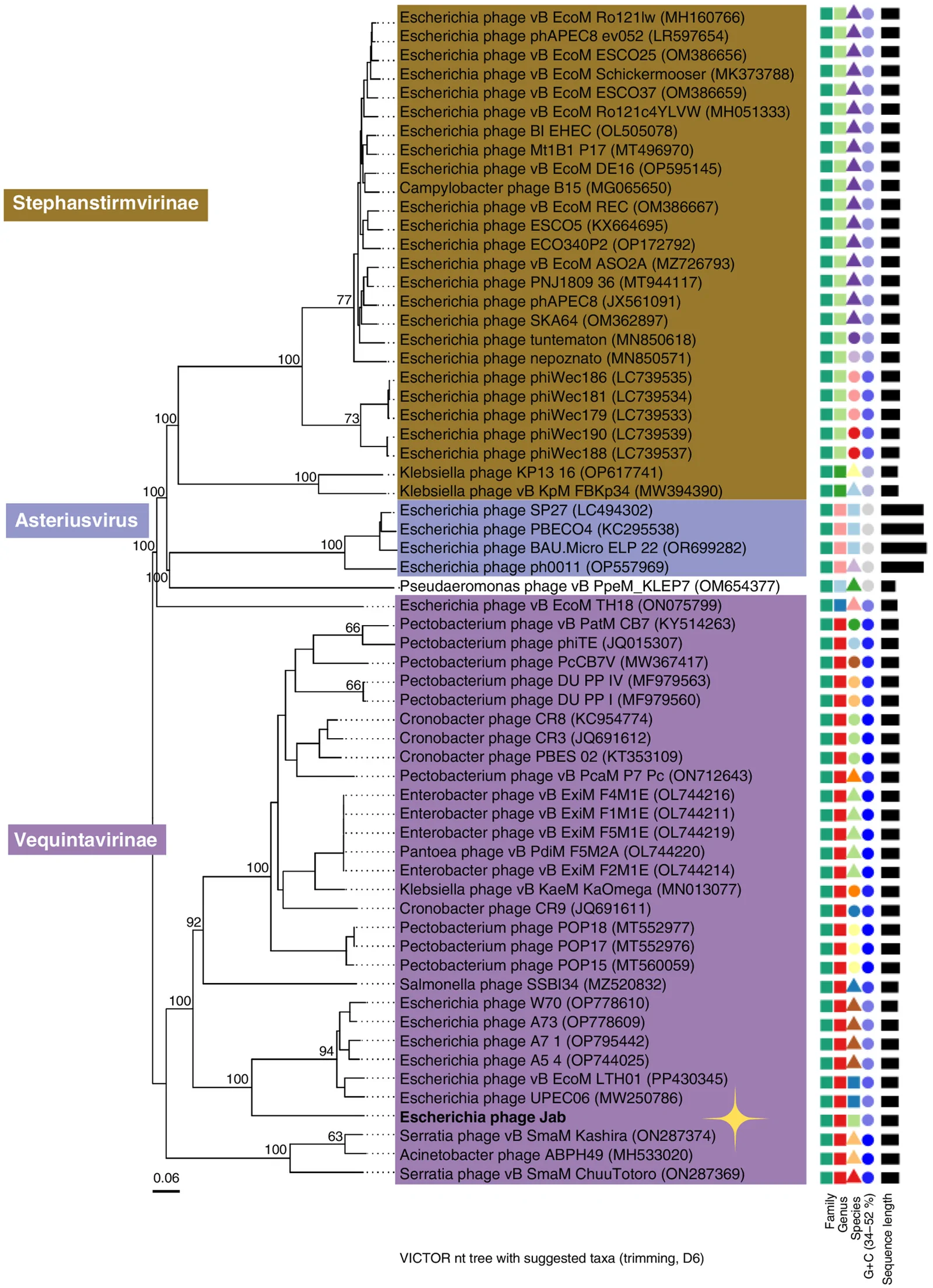
Phylogeny of phage Jab: the GBDP tree is based on complete nucleotide sequences of phage Jab and closely related phages according to vConTACT2 and BLASTn top hits. The numbers above branches are GBDP pseudo-bootstrap support values from 100 replications. Clades containing phages from the same subfamily are color-coded accordingly. The taxonomic information was obtained from GenBank. The phages are clustered into one family, six genera, and 26 species. The size of the phage genomes is displayed in the horizontal black lines on the right.

**Figure 4:**
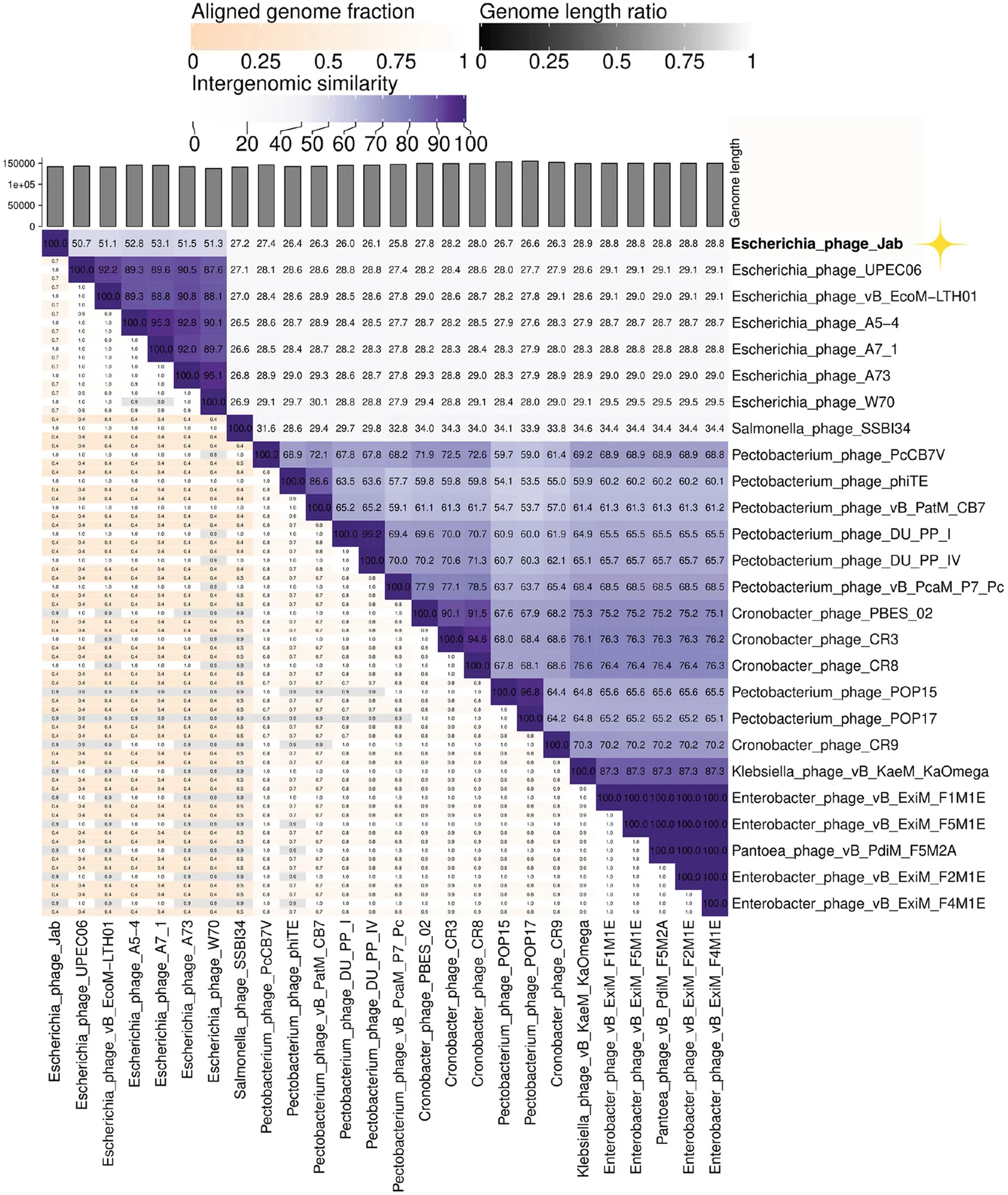
Intergenomic similarity between phage Jab and the most similar phages according to vConTACT2, calculated using VIRIDIC. The heat map indicates genome similarity. Accession numbers of displayed phages are shown in Supplementary Table S4.

**Figure 5:**
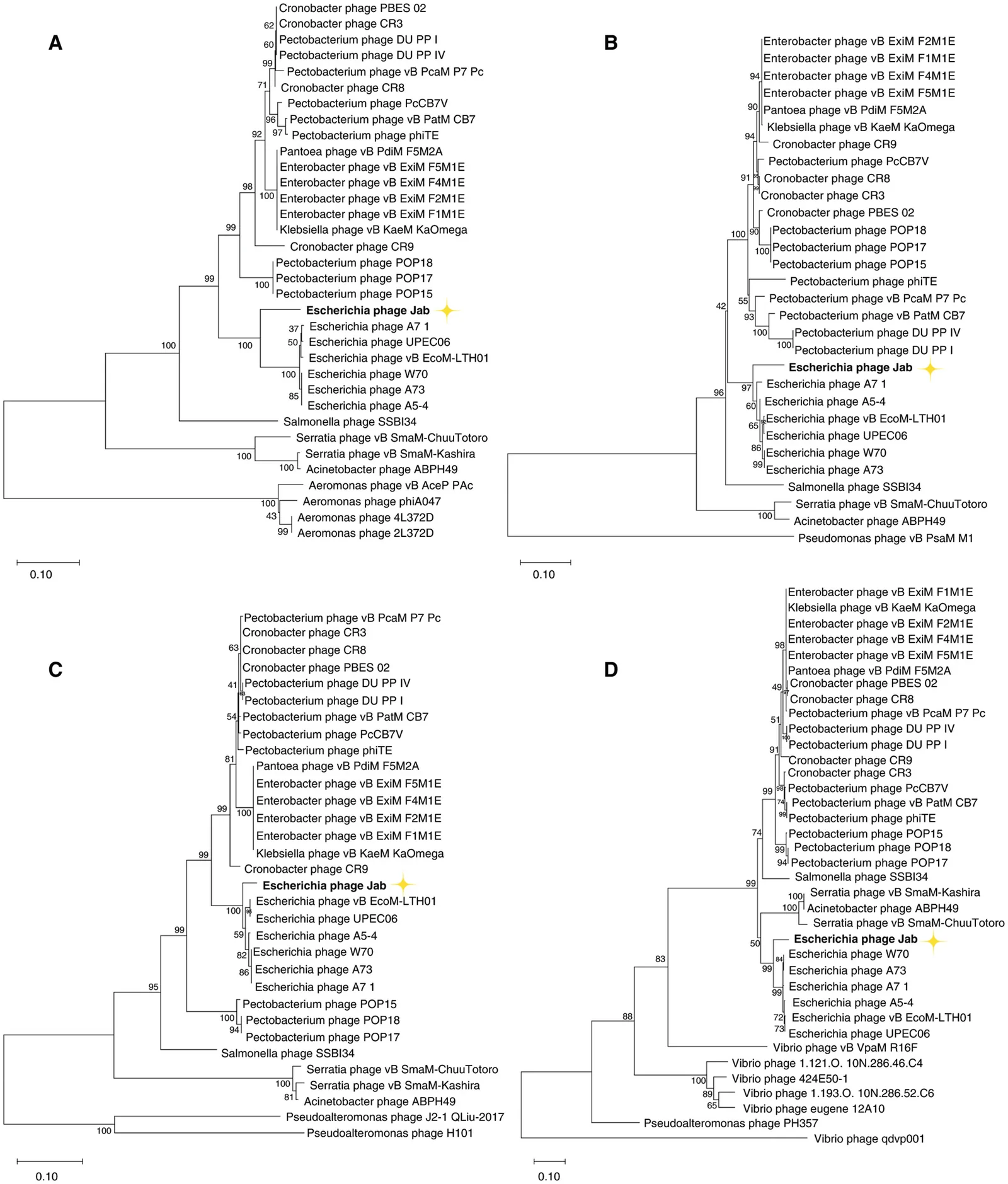
Phylogenetic trees based on viral conserved signature proteins. Phages included in the evolutionary analysis were selected based on the identified homologs derived from the corresponding viral protein clusters built by vConTACT2. Depicted are the evolutionary relationships of **(A)** the helicase, **(B)** the DNA polymerase, **(C)** the major capsid protein, and **(D)** the terminase large subunit of phage Jab with the corresponding proteins of selected phages. Accession numbers of displayed phages are shown in Supplementary Table S4.

The topology of the VICTOR tree showed that phage Jab groups within the phylogenetic radiation of *Vequintavirinae* with high bootstrap values supporting a common phylogenetic ancestor with members of the newly introduced *Septuagintavirus* (Figure 3). The Virus Intergenomic Distance Calculator (VIRIDIC) analysis confirmed that phage Jab had the highest genomic similarity with members of this novel genus; however, with values ranging only from 50% to 53% (Figure 4). Furthermore, the genomic distance to other recognized genera within the *Vequintavirinae* was less than 30% (Figure 4). Those values are well below the International Committee on Taxonomy of Viruses (ICTV) threshold of 95% similarity for the identification of a new virus species and also below the cutoff value of 70% similarity for the identification of a new virus genus (Turner et al., 2021). In addition, the amino acid alignment and phylogenetic treeing analysis of four signature genes, encoding for a helicase (ORF 201), a DNA polymerase (ORF 169), a major capsid protein (ORF 118), and a terminase (ORF 110, 113), consistently confirmed that phage Jab groups closest but distinct to *Septuagintavirus* (Figure 5). Here, the major capsid protein and terminase exhibited the highest similarity values (over 90%), while the DNA polymerase and the helicase showed similarity values below 90 and 80%, respectively.

**Figure 6:**
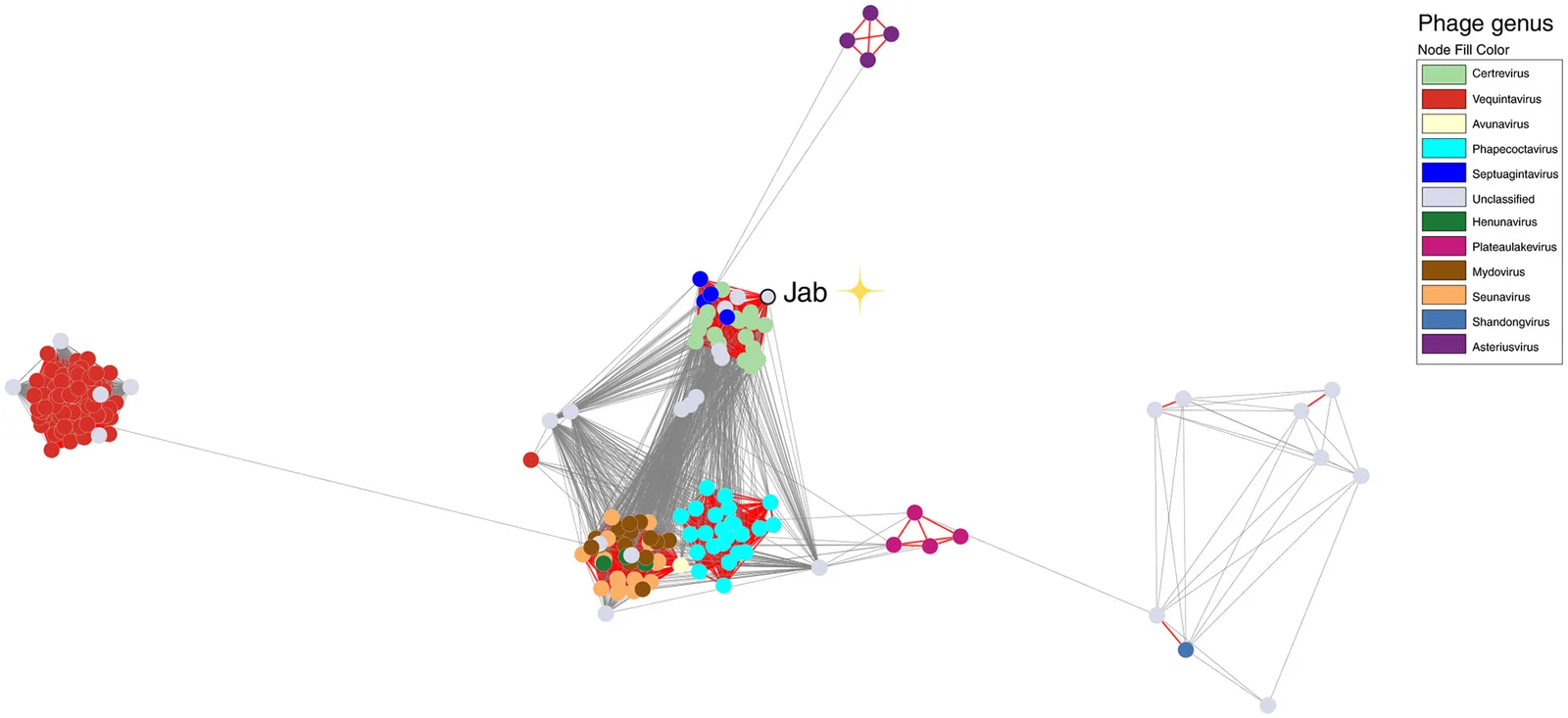
Protein-sharing network indicating evolutionary affinity among Escherichia phage Jab and its related phages. Each node represents a phage genome and is colored according to its genus. The node of Escherichia phage Jab is outlined in black. Edges connecting pairwise phages from the same viral cluster (pre_VC), as determined by vConTACT2, are illustrated in bold and colored red. In addition to Escherichia phage Jab, the species names of the phages with an “unclassified” genus are the following: two members of the subfamily *Stephanstirmvirinae*, Klebsiella phages KP13-16 and vB_KpM_FBKp34; members of the subfamily *Ounavirinae*, Pseudoalteromonas phages J2-1_QLiu-2017 and PH357, Pseudaeromonas phage vB_PpeM_ KLEP7, Pseudomonas phage vB_PaeM_M1; and members of the subfamily *Vequinatvirinae*, Campylobacter phages C5, C11, C14, A116, A138, Salmonella phages 40 and SSBI34, Vibrio phages eugene, qdvp001, 1.193.O._10N.286.52.C6, 1.121.O._10N.286.46.C4, vB_VpaM_R16F and 424E50-1, Klebsiella phage KP12, Acinetobacter phage ABPH49, Pectobacterium phage POP18, Serratia phages vB_SmaM-ChuuTotoro and vB_SmaM-Kashira, and Escherichia phages UPEC06, vB_EcoM_TH18 and vB_EcoM-LTH01.

**Figure 7:**
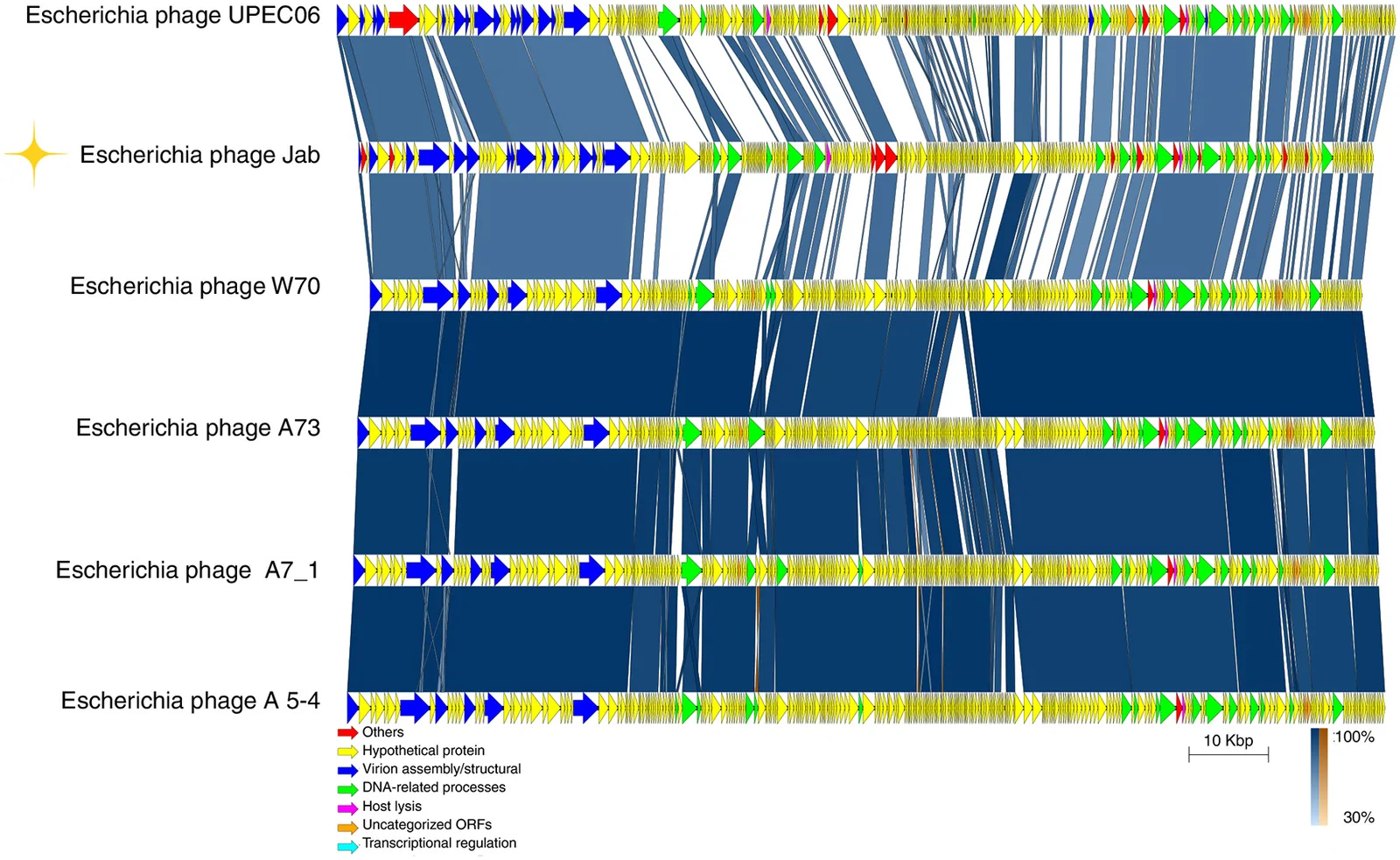
Genome map comparison between the genomes of Escherichia phage Jab isolated in this study, Escherichia phage UPEC06 and members of the genus *Septuagintavirus*, Escherichia phage W70, A73, A7_1 and A5-4. The map was made with Easyfig and illustrates orange arrows indicating the location and orientation of ORFs among the phage genomes. Vertical lines between genome sequences indicate regions of shared similarity, shaded according to tBLASTx (blue for matches in the same direction or orange for inverted matches). The first gene among all genomes was set at the terminase large subunit.

Phage Jab was classified into Viral Cluster 981 by vConTACT, based on MCL clustering, together with 26 other phages, namely phages of the genus *Certrevirus* and *Septuagintavirus* (Figure 6). However, Escherichia phage Jab was classified as a singleton by vContact2 in the subclustering process by ClusterONE (subcluster 981.2, see Supplementary Table S3). Subclusters are considered to be the equivalent of genus grouping. Thus, these results suggest that phage Jab likely represents a new genus. Significant gene synteny was observed between Escherichia phage Jab and UPEC06 and between Escherichia phage Jab and Escherichia phage W70 (Figure 7).

In conclusion, whole-genome nucleotide identity as depicted by the VICTOR tree indicates the distinctiveness of phage Jab at the taxonomic level, while VIRIDIC provides an alignment-based genome similarity, where values below 70% are generally regarded to support separation into a different genus. In addition, gene-sharing network analysis examines how genomes are connected based on shared protein families. Taken together, these analyses provide complementary evidence, and when pointing in the same direction, there is stronger evidence for a putative novel genus.

### 3.3 Phenotypic characteristics

#### 3.3.1 Determination of plaque and phage morphology

**Figure 8:**
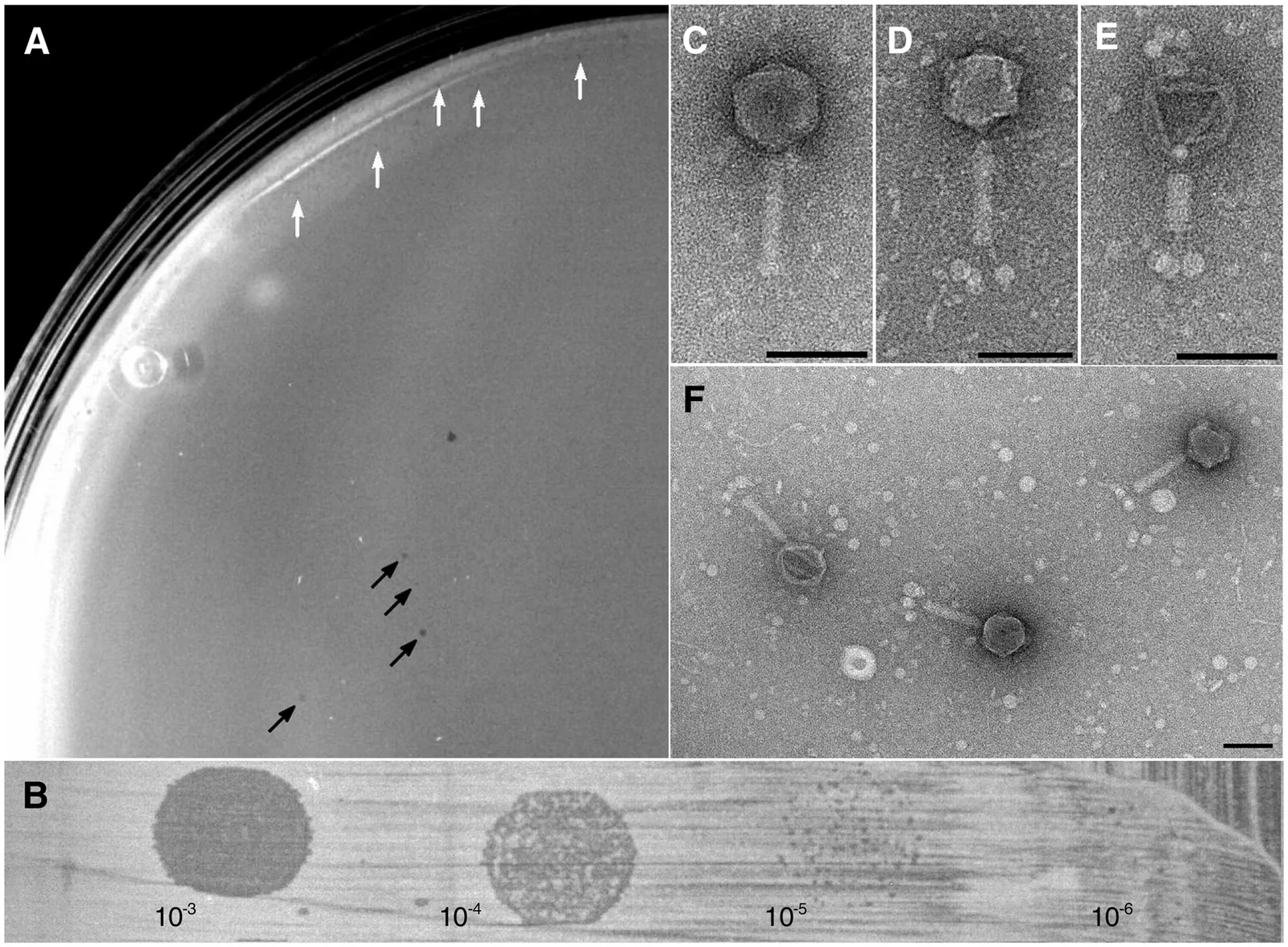
Biological features of phage Jab. **(A)** Plaques of phage Jab on the lawn of *E. coli* ECH07 isolate. **(B)** Plaque assay showing infection of *E. coli* ECH07. Ten-fold serial dilutions of phages (10^-3 to 10^-6) were spotted from left to right onto the bacterial host lawns. **(C–F)** Transmission electron microscopy (TEM) images of the isolated phage Jab. The scale bars in each TEM image represent 100 nm. **(D–F)** show vesicles bound to the tail fibers of phage Jab. In **(E)** the phage tail is contracted. TEM images indicate that phage Jab has the classic shape of myoviruses with a contractile tail.

On solid medium, phage Jab produced relatively small plaques with a diameter of approximately 0.5 mm (Figures 8A,B). Clear zones were observed over a bacterial lawn due to the lytic activity of the phage. On double-layer agar plates, the visibility of the plaques was more pronounced at the edge of the plate, possibly due to the thinner layer of soft agar in that region (Figure 8A). TEM analysis revealed that the phage Jab had an icosahedric capsid of 99.78±3.32 nm (length), 88.9±2.42 nm (width) and a long contractile tail with 127.97±2.89 nm (length) 22.43±1.17 nm (width), which are typical morphological characteristics of myoviruses (Figures 8C–F). When propagated in another host strain, *E. coli* BL21, the TEM image showed 3–4 distinct spherical structures with a size of 20.8–36.1 nm, which were attached to the tail fibers of phage Jab (Figures 8D–F). It was also possible to observe phage Jab with a contracted tail when bound to the spherical structures (Figure 8E). Tail fiber spherical structures were observed in TEM images from two independently generated phage preparations. No such spherical structures could be observed with TEM when phage Jab was propagated with ECH07 (Figure 8C).

To evaluate whether those structures represent outer membrane vesicles (OMVs) produced by the host, external bacterial products (including putative OMVs) were isolated from culture supernatants of *E. coli* strain BL21 and ECH07 and subsequently incubated with phage Jab. However, TEM did not reveal any physical contact between the phage and the putative OMVs, irrespective of their strain origin.

#### 3.3.2 Host range

In this study, the host range of phage Jab was tested against 44 clinical pathogenic and laboratory strains, including 27 *E. coli* strains, and strains of *Pseudomonas aeruginosa, Klebsiella pneumoniae* and *Serratia marcescens*. Host range testing revealed that phage Jab was specific to *E. coli* strains and could not infect any strains of other bacterial species tested. However, phage Jab could infect 8 of the 27 tested *E. coli* strains. Among these were 4 phage-sensitive reference strains (DSMZ 30083, DSMZ 613, BL21, RP437) and 4 phage-sensitive clinical strains. Resistance to phage infection was observed in 4 reference strains (DSMZ 101113, DSMZ 4776, DSMZ 498, and DSMZ 540) and 14 clinical strains. In reference strain DSMZ 5695, lysis from without was observed with no evidence of productive infection. The highest efficiency of plating (EOP) was seen in *E. coli* strain BL21 (Table 1).

**Table 1:** Host range and EOP of phage Jab; + productive infection, – no sensitivity, (–) lysis from without.

Bacterial host	Strain no	Infectivity	EOP
*E. coli*	Clinical strains ECH 01-06, 08-13, 15, 17	–	0
	Clinical strain ECH 07	+	0.06
	Clinical strain ECH 14	+	0.04
	Clinical strain ECH 16	+	0.04
	Clinical strain ECH 18	+	0.59
	DSMZ 101113	–	0
	DSMZ 4776	–	0
	DSMZ 498	–	0
	DSMZ 5695	(–)	0
	DSMZ 540	–	0
	DSMZ 30083	+	0.02
	DSMZ 613	+	0.002
	RP437	+	0.95
	BL21	+	1
*Klebsiella pneumoniae*	KP01, KP02, KPH01, KPH02, KPH03	–	0
	DSMZ 9377, 2026	–	0
*Serratia marcescens*	SM01-SM06	–	0
	DSMZ 30121, 1636	–	0
*Pseudomonas aeruginosa*	DSMZ 22644, 5007	–	0

## 4 Discussion

### 4.1 Looking for a needle in a haystack

In this study, we characterized a novel *E. coli* phage likely representing a hitherto undescribed genus. Typically, the discovery of new phages results from a standard workflow with the following sequence: phage enrichment, isolation, and identification. The phages that are ultimately isolated are therefore initially unknown or essentially left to chance. Conversely, here, we first characterized the genomic diversity of an enriched phage community and then pursued a targeted isolation of phage Jab. Based on the conventional workflow, this phage would have remained elusive due to the overwhelming abundance of the competing phages Twora and Foura in the initial enrichment. Instead, enriched phage lysates were recycled for iterative enrichment rounds, whereby the proportion of phage Jab (the “needle in a haystack”) could be gradually increased. The core concept was to enable the bacterial host ECH07 to evolve resistance against phages Twora and Foura. However, resistance against one phage abolished the previously obtained resistance against the other phage, such that phage Twora and Foura alternatively became numerically dominant in the following enrichment rounds. Apparently, sequential exposure failed to “train” ECH07 to develop dual-phage resistance—a bacterial response previously documented by Wright et al. (2019). Alternatively, simultaneous exposure of ECH07 to both phages could have elicited a generalized resistance, as described earlier in Betts et al. (2016). However, we did not pursue this strategy further, as host switching to *E. coli* BL21 during the last two enrichment rounds proved successful. Notably, phage Jab could not be efficiently propagated based on multiple enrichment rounds using BL21 only (data not shown). Instead, ultimate isolation was only possible by leaving phage Foura behind (i.e., propagation with a Foura-resistant host), and subsequent switch to BL21, where Jab outcompeted the remaining two phages Twora and Threena (Figure 1). Thus, the acquisition and loss of resistance, precise timing of host switching, and fluctuating phage compositions collectively governed the phage competition outcomes—which seem inherently unpredictable.

### 4.2 Taxonomic characterization of phage Jab

Genomic analysis of phage Jab revealed that this phage fulfills the taxonomic criteria for the assignment to a putative new genus. This was borne out by the data from the VIRIDIC analysis as well as by the content-based network analysis. Since Escherichia phage Jab was classified into the same viral cluster by vContact2 as phages from the *Certrevirus* and *Septuagintavirus* genus but not the same subcluster, it is highly likely that Escherichia phage Jab is related to them at roughly the genus-subfamily level. Further support for its distinctiveness is provided by the topological position within the VICTOR tree, where phage Jab groups in proximity but distinct from members of the newly proposed genus *Septuagintavirus*. Interestingly, the VICTOR analysis does not classify phage Jab as a novel genus (see red squares on the right side of Figure 3). However, differences between VIRIDIC and VICTOR analysis have been reported for taxonomic assignments at genus levels, and likely arise because VIRIDIC uses fixed similarity cutoffs (70% genus), while VICTOR emphasizes tree-based monophyly (Kauffman et al., 2022; O'Connell et al., 2024). Notably, VICTOR analysis also wrongly classified the recognized genera *Certrevirus* and *Septuagintavirus* as one genus (Figure 3), which shows that VICTOR tends to group more broadly at the genus level, making VIRIDIC and network analysis probably more suitable for genus classification. Independent support for the distinctiveness of phage Jab is provided by an OrthoVenn3 analysis (Sun et al., 2023). When comparing orthologous gene clusters between Jab and the four members of the nearest taxonomic genus *Septuagintavirus* (i.e., phages W40, A7-1, A73, and SV54), using default similarity cutoffs, OrthoVenn identified 110 proteins as singletons (unique proteins not shared with the other phages in the dataset). 80% of them (88 genes) were found in the genome of Jab, signifying the genomic novelty of this phage. In addition, while gene synteny is highly conserved across the four members of *Septuagintavirus*, the genome map comparison shows that the overall arrangement of the genome of Jab lies between *Septuagintavirus* and the unclassified phage UPEC06 (Figure 7). Nevertheless, since the four independent marker proteins all place phage Jab together with the members of *Septuagintavirus*, this strongly suggests a common evolutionary ancestry as well as a shared DNA replication strategy and packaging machinery (Hyde et al., 2024).

### 4.3 Salient genomic and phenotypic features of phage Jab

In the genome of phage Jab, less than 20% of genes encode proteins with putatively known functions. This is somewhat lower compared to members of the *Septuagintavirus*, where a functional assignment was possible for 26% of genes (Cortés-Martín et al., 2024). Best hits for genes with functional annotation were mostly found with *Septuagintavirus* or with the unclassified phage UPEC06 (Kazibwe et al., 2022) and largely represent proteins with common functions for members within the *Caudoviricetes*. Interestingly, in addition to an aerobic class I ribonucleotide reductase (RNR), the genome of phage Jab also encodes an anaerobic class III ribonucleotide reductase (RNR), shared by the members of the *Septuagintavirus*. This characteristic appears to be a hallmark feature of members of the *Vequintavirinae*, although this enzyme is not universally present in this subfamily (Cortés-Martín et al., 2024). Otherwise, class III RNRs are relatively rare in phage genomes, accounting for around 28% in ribonucleotide reductase-positive phages and less than 5% in all sequenced phages (Dwivedi et al., 2013). Class III RNRs enable deoxynucleoside triphosphate (dNTP) synthesis under oxygen-limited conditions during infection, compensating for host RNR shutdown or hypoxia in natural niches (Hernández Villamizar et al., 2023). Thus, the oxygen-poor source environment of phage Jab (i.e., manure or the animal gut, respectively) likely selects for phages that have acquired class III RNRs via horizontal gene transfer (Dwivedi et al., 2013). This in turn might imply that phages equipped with class III RNRs could be useful for therapy of hypoxic infections, caused by facultative anaerobes, such as *E. coli* (André et al., 2021).

In the genome of phage Jab, we found four genes encoding putative depolymerases. These enzymes are of great interest as they can aid in the degradation of bacterial biofilms (Guo et al., 2023). So far, depolymerases of only a comparably small proportion of *E. coli* phages have been experimentally characterized (Knecht et al., 2020; Pires et al., 2016). Those investigations indicated that the genomes of *E. coli* phages typically encode one or two depolymerases (Knecht et al., 2020), but in a few cases also up to four depolymerases (Plattner et al., 2019), making the existence of four functional depolymerases in phage Jab at least theoretically possible. However, although new *E. coli* phages are continually being isolated and sequenced, the majority of the predicted depolymerases have so far been annotated only through bioinformatics. Given the high number of *E. coli* capsular types (Whitfield, 2009), a likewise high diversity of phage-encoded depolymerases remains to be identified and functionally verified. Therefore, future studies should clone and express the four genes in question for experimental verification. Determining the specificity of these depolymerases–including the putative enzymes from phage Jab targeting capsular, exo-, or lipopolysaccharides–is of utmost importance.

Although members of the subfamily *Vequintavirinae* are characterized by a comparably large host range (Maffei et al., 2021; Humolli et al., 2025), the phages of the newly described genus *Septuagintavirus* display an extremely narrow host range (Cortés-Martín et al., 2024). For instance, the phage W70, which is most closely related to phage Jab, could infect only one clinical strain out of 16 tested. In contrast, phage Jab could infect four out of 14 clinical strains, which may be due to the possible existence of four functional depolymerases, which typically enable the infection of multiple capsule types (Wu et al., 2023; Pan et al., 2017).

As a limitation of this study, phenotypic characteristics of phage Jab, such as adsorption rate, one-step growth analysis, burst size, antibacterial potential in liquid infection assays or biofilms, as well as stability testing, were not further investigated, as the primary objective was the targeted isolation and genomic characterization. Therefore, further experiments are needed to fully understand the biological properties and application potential.

One peculiar feature of phage Jab is the occurrence of small spherical structures attached at the end of the tail fibers. One could assume that the observed structures are outer membrane vesicles (OMVs) originating from the *E. coli* strain BL21, as they could not be observed when phage Jab was propagated with the *E. coli* strain ECH07. OMVs produced by Gram-negative bacteria typically range from 20-200 nm in size, and evidence suggests that they act as a defense strategy against phages (Reyes-Robles et al., 2018; Manning and Kuehn, 2011; Stephan et al., 2020). In this regard, OMVs may function as phage decoys, reducing infection efficiency. By physically interfering with predatory phages, bacteria may reduce the need for evolving genetic resistance, which is often associated with fitness costs (Tang et al., 2023; Landor et al., 2024). The role of OMVs as effective phage protection has been demonstrated for *E. coli* strains previously, but not so far for BL21 (Manning and Kuehn, 2011; Xuan et al., 2024). It is known, however, that BL21 is a strong OMV producer (Sahu et al., 2025). Therefore, further experimentation is warranted to assess the identity and function of those spherical structures, for example, by lipid staining, proteomic characterization, cryo-TEM, or by immunogold labeling. In any case, even if the observed structures interfere negatively with phage adsorption, apparently this was not effective enough to halt infectivity of phage Jab. Importantly, we are not aware of any study describing multiple 20–30 nm sized spherical structures, each of which attached to a single tail fiber, as was seen in the TEM pictures of phage Jab.

### 4.4 Broadening our view of phage diversity through targeted isolation

While the number of well-characterized and taxonomically classified phages is constantly increasing, the diversity of described phages within subfamilies is unequally covered. According to the International Committee on Taxonomy of Viruses (ICTV) and taking two subfamilies of the *Caudoviricetes* as an example, the subfamily *Tevenvirinae* (family *Straboviridae*) currently consists of 14 different genera comprising 148 species, of which 85 phage species belong to the genus *Tequatrovirus* (Black et al., 2026). The remaining 13 genera are represented by a range of one to a maximum of 15 species only. Likewise, the subfamily *Vequintavirinae* (no family assignment) consists of seven recognized genera comprising 56 species with 36 species belonging to the genus *Vequintavirus* (Black et al., 2026). The remaining six genera are again represented by a few species only, ranging from one to a maximum of six species.

Although the phylogenetic depth and diversity might differ between those two viral subfamilies, some evidence of a preferential retrieval of *Tevenvirinae* phages compared to *Vequintavirinae* phages from environmental sources exists as well. For instance, to enlarge the known diversity of *E. coli* phages, 35 new *Caudoviricetes* were isolated and characterized recently, based on different environmental sources and 29 different *E. coli* strains as hosts. Remarkably, 27/35 phages (77%) belonged to the *Tevenvirinae* subfamily, whereas only 2/35 phages belonged to the *Vequintavirinae* subfamily (Korf et al., 2019). In addition, in another study, also based on various environmental sources and 19 genetically distinct *E. coli* strains as hosts, 28 new phages were isolated, of which again the majority (i.e., 15 phages = 83%) belong to *Tevenvirinae*. Only one out of the 28 new phages belonged to *Vequintavirinae* (genus *Mydovirus*). Notably, this phage was obtained by direct plating of the environmental sample on the bacterial lawn without prior propagation, indicative of its comparably high numerical abundance in the original source (Vitt et al., 2024).

This unequal representation of phage genera and species in databases, both within and between subfamilies, likely does not reflect the true composition of the environmental phage communities, but instead results from preferential amplification, in which established phage types outpace others during laboratory enrichment procedures. This is also borne out by the results of a recent study, in which unbiased phage propagation was achieved through creation of single-droplet microfluidic-based environments, where phages had equal chances of host infection (Zhang et al., 2026). Besides rivalry for the same host, isolation of phages is further biased towards those being able of plaque formation, resulting in the complete neglect of non-plaque-forming phages (Zhang et al., 2026; Friedrich et al., 2023).

Therefore, it is a promising avenue for future work to dissect the phage populations within enrichments, for example, by metagenomic analyses, to track down and isolate underexplored phages. Moreover, the controlled alteration of physicochemical enrichment parameters may shift the selective advantage toward phages that are notoriously overlooked because of their inferiority during conventional approaches. We hypothesize that deliberately reversing competitive dominance among phages will reveal a broader and currently underappreciated spectrum of phages that can be isolated in the laboratory.

## Data Availability

The datasets presented in this study can be found in online repositories. The names of the repository/repositories and accession number(s) can be found in the article/Supplementary material.

## References

[B1] André A. C. Debande L. Marteyn B. S. (2021). The selective advantage of facultative anaerobes relies on their unique ability to cope with changing oxygen levels during infection. Cell. Microbiol. 23:e13338. doi: 10.1111/cmi.13338 33813807

[B2] Besemer J. Lomsadze A. Borodovsky M. (2001). Genemarks: a self-training method for prediction of gene starts in microbial genomes. Implications for finding sequence motifs in regulatory regions. Nucleic Acids Res. 29, 2607–2618. doi: 10.1093/nar/29.12.2607 11410670 PMC55746

[B3] Betts A. Gifford D. R. MacLean R. C. King K. C. (2016). Parasite diversity drives rapid host dynamics and evolution of resistance in a bacteria-phage system. Evolution 70, 969–978. doi: 10.1111/evo.12909 27005577 PMC4982092

[B4] Bin Jang H. Bolduc B. Zablocki O. Kuhn J. H. Roux S. Adriaenssens E. M. . (2019). Taxonomic assignment of uncultivated prokaryotic virus genomes is enabled by gene-sharing networks. Nat. Biotechnol. 37, 632–639. doi: 10.1038/s41587-019-0100-8 31061483

[B5] Black E. J. Powell C. S. Dempsey D. M. Hendrickson R. C. Mims L. R. Lefkowitz E. J. (2026). Virus taxonomy: the database of the international committee on taxonomy of viruses. Nucleic Acids Res. 54, D776-D789. doi: 10.1093/nar/gkaf1159 41296552 PMC12807731

[B6] Cook R. Brown N. Redgwell T. Rihtman B. Barnes M. Clokie M. . (2021). Infrastructure for a phage reference database: identification of large-scale biases in the current collection of cultured phage genomes. Phage 2, 214–223. doi: 10.1089/phage.2021.0007 36159887 PMC9041510

[B7] Cortés-Martín A. Buttimer C. Pozhydaieva N. Hille F. Shareefdeen H. Bolocan A. S. . (2024). Isolation and characterization of septuagintavirus; a novel clade of *Escherichia coli* phages within the subfamily vequintavirinae. Microbiol. Spectr. 12:e00592-24. doi: 10.1128/spectrum.00592-24 39101714 PMC11370258

[B8] d'Herelle F. (1926). The Bacteriophage and Its Behavior. Baltimore, MD: Williams & Wilkins.

[B9] Dion M. B. Oechslin F. Moineau S. (2020). Phage diversity, genomics and phylogeny. Nat. Rev. Microbiol. 18, 125–138. doi: 10.1038/s41579-019-0311-5 32015529

[B10] Dwivedi B. Xue B. Lundin D. Edwards R. A. Breitbart M. (2013). A bioinformatic analysis of ribonucleotide reductase genes in phage genomes and metagenomes. BMC Evol. Biol. 13:33. doi: 10.1186/1471-2148-13-33 23391036 PMC3653736

[B11] Friedrich I. Neubauer H. Kuritsyn A. Bodenberger B. Tskhay F. Hartmann S. . (2023). Brevundimonas and serratia as host systems for assessing associated environmental viromes and phage diversity by complementary approaches. Front. Microbiol. 14:1095850. doi: 10.3389/fmicb.2023.1095850 37025643 PMC10070969

[B12] Göker M. García-Blázquez G. Voglmayr H. Tellería M. T. Martín M. P. (2009). Molecular taxonomy of phytopathogenic fungi: a case study in peronospora. PLoS One 4:e6319. doi: 10.1371/journal.pone.0006319 19641601 PMC2712678

[B13] Guo Z. Liu M. Zhang D. (2023). Potential of phage depolymerase for the treatment of bacterial biofilms. Virulence 14:2273567. doi: 10.1080/21505594.2023.2273567 37872768 PMC10621286

[B14] Hernández Villamizar S. Chica Cárdenas L. A. Morales Mancera L. T. Vives Florez M. J. (2023). Anaerobiosis, a neglected factor in phage-bacteria interactions. Appl. Environ. Microbiol. 89:e01491-23. doi: 10.1128/aem.01491-23 37966212 PMC10734468

[B15] Hockenberry A. J. Wilke C. O. (2021). BACPHLIP: predicting bacteriophage lifestyle from conserved protein domains. PeerJ 9:e11396. doi: 10.7717/peerj.11396 33996289 PMC8106911

[B16] Humolli D. Piel D. Maffei E. Heyer Y. Agustoni E. Shaidullina A. . (2025). Completing the basel phage collection to unlock hidden diversity for systematic exploration of phage-host interactions. PLoS Biol. 23:e3003063. doi: 10.1371/journal.pbio.3003063 40193529 PMC11990801

[B17] Hyde J. R. Armond T. Herring J. A. Hope S. Grose J. H. Breakwell D. P. . (2024). Diversity and conservation of the genome architecture of phages infecting the alphaproteobacteria. Microbiol. Spectr. 12:e02827-23. doi: 10.1128/spectrum.02827-23 37991376 PMC10783043

[B18] Hyman P. (2019). Phages for phage therapy: isolation, characterization, and host range breadth. Pharmaceuticals 12:35. doi: 10.3390/ph12010035 30862020 PMC6469166

[B19] Kauffman K. M. Chang W. K. Brown J. M. Hussain F. A. Yang J. Polz M. F. . (2022). Resolving the structure of phage-bacteria interactions in the context of natural diversity. Nat. Commun. 13:372. doi: 10.1038/s41467-021-27583-z 35042853 PMC8766483

[B20] Kazibwe G. Ndekezi C. Alinaitwe R. Alafi S. Nanteza A. Kimuda M. P. . (2022). Genome sequences of bacteriophages UPEC01, UPEC03, UPEC06, and UPEC07 infecting avian pathogenic *Escherichia coli. Microbiol. Resour. Announc*. 11:e00811-21. doi: 10.1128/mra.00811-21 35262399 PMC8928767

[B21] Knecht L. E. Veljkovic M. Fieseler L. (2020). Diversity and function of phage encoded depolymerases. Front. Microbiol. 10:2949. doi: 10.3389/fmicb.2019.02949 31998258 PMC6966330

[B22] Kohl M. Wiese S. Warscheid B. (2011). “Cytoscape: software for visualization and analysis of biological networks,” in Data Mining in Proteomics: From Standards to Applications, eds. M. Hamacher, M. Eisenacher, and C. Stephanpages (Totowa, NJ: Humana Press), 291–303. doi: 10.1007/978-1-60761-987-1_18 21063955

[B23] Korf I. H. Meier-Kolthoff J. P. Adriaenssens E. M. Kropinski A. M. Nimtz M. Rohde M. . (2019). Still something to discover: novel insights into *Escherichia coli* phage diversity and taxonomy. viruses 11:454. doi: 10.3390/v11050454 31109012 PMC6563267

[B24] Kumar S. Stecher G. Tamura K. (2016). MEGA7: molecular evolutionary genetics analysis version 7.0 for bigger datasets. Mol. Biol. Evol. 33, 1870–1874. doi: 10.1093/molbev/msw054 27004904 PMC8210823

[B25] Landor L. A. Tjendra J. Erstad K. Krabberød A. K. Töpper J. P. Våge S. (2024). At what cost? The impact of bacteriophage resistance on the growth kinetics and protein synthesis of *Escherichia coli. Environ. Microbiol. Rep*. 16:e70046. doi: 10.1111/1758-2229.70046 39562842 PMC11576411

[B26] Lefort V. Desper R. Gascuel O. (2015). FastME 2.0: a comprehensive, accurate, and fast distance-based phylogeny inference program. Mol. Biol. Evol. 32, 2798–2800. doi: 10.1093/molbev/msv150 26130081 PMC4576710

[B27] Maffei E. Shaidullina A. Burkolter M. Heyer Y. Estermann F. Druelle V. . (2021). Systematic exploration of *Escherichia coli* phage-host interactions with the basel phage collection. PLoS Biol. 19:e3001424. doi: 10.1371/journal.pbio.3001424 34784345 PMC8594841

[B28] Magill D. J. Skvortsov T. A. (2023). DePolymerase predictor (DePP): a machine learning tool for the targeted identification of phage depolymerases. BMC Bioinform. 24:208. doi: 10.1186/s12859-023-05341-w 37208612 PMC10199479

[B29] Manning A. J. Kuehn M. J. (2011). Contribution of bacterial outer membrane vesicles to innate bacterial defense. BMC Microbiol. 11:258. doi: 10.1186/1471-2180-11-258 22133164 PMC3248377

[B30] Meier-Kolthoff J. P. Auch A. F. Klenk H.-P. Göker M. (2013). Genome sequence-based species delimitation with confidence intervals and improved distance functions. BMC Bioinform. 14, 1–14. doi: 10.1186/1471-2105-14-60 23432962 PMC3665452

[B31] Meier-Kolthoff J. P. Göker M. (2017). VICTOR: genome-based phylogeny and classification of prokaryotic viruses. Bioinformatics 33, 3396–3404. doi: 10.1093/bioinformatics/btx440 29036289 PMC5860169

[B32] Meier-Kolthoff J. P. Hahnke R. L. Petersen J. Scheuner C. Michael V. Fiebig A. . (2014). Complete genome sequence of DSM 30083(T), the type strain (U5/41(T)) of *Escherichia coli*, and a proposal for delineating subspecies in microbial taxonomy. Stand. Genomic Sci. 9, 1–19. doi: 10.1186/1944-3277-9-2 25780495 PMC4334874

[B33] Moraru C. Varsani A. Kropinski A. M. (2020). Viridic–a novel tool to calculate the intergenomic similarities of prokaryote-infecting viruses. Viruses 12:1268. doi: 10.3390/v12111268 33172115 PMC7694805

[B34] Nayfach S. Camargo A. P. Schulz F. Eloe-Fadrosh E. Roux S. Kyrpides N. C. (2021). CheckV assesses the quality and completeness of metagenome-assembled viral genomes. Nat. Biotechnol. 39, 578–585. doi: 10.1038/s41587-020-00774-7 33349699 PMC8116208

[B35] Nurk S. Meleshko D. Korobeynikov A. Pevzner P. A. (2017). metaSPAdes: a new versatile metagenomic assembler. Genome Res. 27, 824–834. doi: 10.1101/gr.213959.116 28298430 PMC5411777

[B36] O'Connell L. M. Coffey A. O'Mahony J. M. (2024). Genomic analysis of seven mycobacteriophages identifies three novel species with differing phenotypic stabilities. Heliyon 10:e27932. doi: 10.1016/j.heliyon.2024.e27932 38515691 PMC10955285

[B37] Paez-Espino D. Eloe-Fadrosh E. A. Pavlopoulos G. A. Thomas A. D. Huntemann M. Mikhailova N. . (2016). Uncovering earth's virome. Nature 536, 425–430. doi: 10.1038/nature19094 27533034

[B38] Pan Y.-J. Lin T.-L. Chen C.-C. Tsai Y.-T. Cheng Y.-H. Chen Y.-Y. . (2017). Klebsiella phage ϕk64-1 encodes multiple depolymerases for multiple host capsular types. J. Virol. 91, 10–1128. doi: 10.1128/JVI.02457-16 PMC5331798 28077636

[B39] Peirano G. Pitout J. D. (2025). Rapidly spreading enterobacterales with OXA-48-like carbapenemases. J. Clin. Microbiol. 63:e01515-24. doi: 10.1128/jcm.01515-24 39760498 PMC11837536

[B40] Pires D. P. Oliveira H. Melo L. D. Sillankorva S. Azeredo J. (2016). Bacteriophage-encoded depolymerases: their diversity and biotechnological applications. Appl. Microbiol. Biotechnol. 100, 2141–2151. doi: 10.1007/s00253-015-7247-0 26767986

[B41] Plattner M. Shneider M. M. Arbatsky N. P. Shashkov A. S. Chizhov A. O. Nazarov S. . (2019). Structure and function of the branched receptor-binding complex of bacteriophage CBA120. J. Mol. Biol. 431, 3718–3739. doi: 10.1016/j.jmb.2019.07.022 31325442

[B42] Pye H. V. Krishnamurthi R. Cook R. Adriaenssens E. M. (2024). Phage diversity in one health. Essays Biochem. 68, 607–619. doi: 10.1042/EBC20240012 39475220 PMC12055037

[B43] Reyes-Robles T. Dillard R. S. Cairns L. S. Silva-Valenzuela C. A. Housman M. Ali A. . (2018). Vibrio cholerae outer membrane vesicles inhibit bacteriophage infection. J. Bacteriol. 200:e00792-17. doi: 10.1128/JB.00792-17 29661863 PMC6040182

[B44] Roux S. Adriaenssens E. M. Dutilh B. E. Koonin E. V. Kropinski A. M. Krupovic M. . (2019). Minimum information about an uncultivated virus genome (MIUViG). Nat. Biotechnol. 37, 29–37. doi: 10.1038/nbt.4306 30556814 PMC6871006

[B45] Roux S. Coclet C. (2026). Viromics approaches for the study of viral diversity and ecology in microbiomes. Nat. Rev. Genet. 27, 32–46. doi: 10.1038/s41576-025-00871-w 40691354

[B46] Sahu S. Koningstein G. Bunduc C. M. van der Wel N. Luirink J. van Ulsen P. (2025). Overproduction of β-barrel outer membrane proteins in *Escherichia coli* BL21 (DE3) induces hypervesiculation. Extracell. Vesicles Circ. Nucl. Acids 6:386. doi: 10.20517/evcna.2025.27 41132503 PMC12540271

[B47] Salisbury A. Tsourkas P. K. (2019). A method for improving the accuracy and efficiency of bacteriophage genome annotation. Int. J. Mol. Sci. 20:3391. doi: 10.3390/ijms20143391 31295925 PMC6678273

[B48] Schneider C. A. Rasband W. S. Eliceiri K. W. (2012). NIH image to ImageJ: 25 years of image analysis. Nat. Methods 9, 671–675. doi: 10.1038/nmeth.2089 22930834 PMC5554542

[B49] Seemann T. (2014). Prokka: rapid prokaryotic genome annotation. Bioinformatics 30, 2068–2069. doi: 10.1093/bioinformatics/btu153 24642063

[B50] Stephan M. S. Broeker N. K. Saragliadis A. Roos N. Linke D. Barbirz S. (2020). In vitro analysis of O-antigen-specific bacteriophage P22 inactivation by *Salmonella* outer membrane vesicles. Front. Microbiol. 11:510638. doi: 10.3389/fmicb.2020.510638 33072001 PMC7541932

[B51] Stothard P. Grant J. R. Van Domselaar G. (2019). Visualizing and comparing circular genomes using the CGView family of tools. Brief. Bioinformatics 20, 1576–1582. doi: 10.1093/bib/bbx081 28968859 PMC6781573

[B52] Sullivan M. J. Petty N. K. Beatson S. A. (2011). Easyfig: a genome comparison visualizer. Bioinformatics 27, 1009–1010. doi: 10.1093/bioinformatics/btr039 21278367 PMC3065679

[B53] Sun J. Lu F. Luo Y. Bie L. Xu L. Wang Y. (2023). OrthoVenn3: an integrated platform for exploring and visualizing orthologous data across genomes. Nucleic Acids Res. 51, W397-W403. doi: 10.1093/nar/gkad313 37114999 PMC10320085

[B54] Tang M. Huang Z. Zhang X. Kong J. Zhou B. Han Y. . (2023). Phage resistance formation and fitness costs of hypervirulent *Klebsiella pneumoniae* mediated by K2 capsule-specific phage and the corresponding mechanisms. Front. Microbiol. 14:1156292. doi: 10.3389/fmicb.2023.1156292 37538841 PMC10394836

[B55] Turner D. Kropinski A. M. Adriaenssens E. M. (2021). A roadmap for genome-based phage taxonomy. Viruses 13:506. doi: 10.3390/v13030506 33803862 PMC8003253

[B56] Unterer M. Khan Mirzaei M. Deng L. (2023). Targeted single-phage isolation reveals phage-dependent heterogeneous infection dynamics. Microbiol. Spectr. 11:e05149-22. doi: 10.1128/spectrum.05149-22 37067443 PMC10269501

[B57] Vieira M. F. Duarte J. Domingues R. Oliveira H. Dias O. (2025). PhageDPO: a machine-learning based computational framework for identifying phage depolymerases. Comput. Biol. Med. 188:109836. doi: 10.1016/j.compbiomed.2025.109836 39951981

[B58] Vitt A. R. Sørensen A. N. Bojer M. S. Bortolaia V. Sørensen M. C. H. Brøndsted L. (2024). Diverse bacteriophages for biocontrol of ESBL-and AmpC-β-lactamase-producing *E. coli. Iscience* 27:108826. doi: 10.1016/j.isci.2024.108826 PMC10844046 38322997

[B59] Whitfield C. (2009). Structure and assembly of *Escherichia coli* capsules. EcoSal Plus 3:10–1128. doi: 10.1128/ecosalplus.4.7.3 26443756

[B60] Wright R. C. Friman V.-P. Smith M. C. Brockhurst M. A. (2019). Resistance evolution against phage combinations depends on the timing and order of exposure. MBio 10, 10–1128. doi: 10.1128/mBio.01652-19 PMC6759759 31551330

[B61] Wu J.-W. Wang J.-T. Lin T.-L. Liu Y.-Z. Wu L.-T. Pan Y.-J. (2023). Identification of three capsule depolymerases in a bacteriophage infecting *Klebsiella pneumoniae* capsular types K7, K20, and K27 and therapeutic application. J. Biomed. Sci. 30:31. doi: 10.1186/s12929-023-00928-0 37210493 PMC10199534

[B62] Xuan G. Lu D. Lin H. Wang Y. Wang J. (2024). Outer membrane vesicle production by *Escherichia coli* enhances its defense against phage infection. Microorganisms 12:1836. doi: 10.3390/microorganisms12091836 39338510 PMC11433858

[B63] Zhang H. Boeckman J. Gupte R. Prejean A. Miller J. Rodier A. . (2026). PRISM: a unified platform for phage isolation and characterization from single-droplet microenvironments. Sci. Adv. 12:eaeb2362. doi: 10.1126/sciadv.aeb2362 41880513 PMC13015883

