# Supplementary material for: Genome-guided isolation and characterization of a novel bacteriophage infecting Escherichia coli reveal a putative new genus
Source: Front Microbiol. 2026 Aug 20;17:1909709. doi: 10.3389/fmicb.2026.1909709 (PMC13538427; doi:10.3389/fmicb.2026.1909709)
Supplement: Supplementary file 1 [file Supplementary_File_1.pdf]

Strain	Phage lysis	AMP	AMS	PIP	PIS	CUR	CPO	CTA	CTZ	IMI	MER	ERT	CIP	LEV	GEN	TRS	TIG
ECH01	-	R	R	R	R	R	R	R	R	S	S	n.d.	R	R	R	R	S
ECH02	-	R	R	R	R	R	R	R	R	S	R	R	R	R	S	S	R
ECH03	-	R	R	R	R	R	R	R	R	R	R	R	R	R	R	R	R
ECH04	-	R	R	R	R	R	R	R	R	S	S	R	R	R	S	R	S
ECH05	-	R	R	R	R	R	R	R	R	S	I	n.d.	R	R	S	S	S
ECH06	-	R	R	R	R	R	R	R	R	S	S	R	S	S	S	S	S
ECH07	+	R	R	R	R	R	R	R	R	S	I	R	S	S	S	S	S
ECH08	-	R	R	R	R	R	R	R	R	S	S	R	S	S	S	R	S
ECH09	-	R	R	R	R	R	R	R	R	S	S	n.d.	R	R	S	S	S
ECH10	-	R	R	R	R	R	R	R	R	S	I	R	R	R	S	R	S
ECH11	-	R	R	R	R	R	R	R	R	R	S	R	S	S	S	S	S
ECH12	-	R	R	R	R	R	R	R	R	S	S	n.d.	R	R	R	S	S
ECH13	-	R	R	R	R	I	S	S	S	S	S	S	S	S	S	S	S
ECH14	+	R	R	R	R	R	R	R	R	S	S	R	S	S	S	S	R
ECH15	-	R	R	R	R	R	R	R	R	S	S	R	S	S	S	S	S
ECH16	+	R	R	R	R	R	R	R	R	I	S	R	S	S	S	R	S
ECH17	-	R	R	R	R	R	R	R	R	S	S	R	S	S	S	S	S
ECH18	+	R	R	R	R	R	R	R	R	R	S	R	S	S	S	S	S

Supplementary Figure S1: Susceptibility of clinical strains (ECH01-ECH18) to infection by phage Jab (clear lysis:+; no lysis: -), and antibiotic resistance profiles. AMP: ampicillin, AMS: ampicillin/sulbactam, PIP: piperacillin, PIS: piperacillin/sulbactam, CUR: cefuroxim, CPO: cefpodoxim, CTA: cefotaxim, CTZ: ceftazidim, IMI: imipenem, MER: meropenem, ERT: ertapenem, CIP: ciprofloxacin; LEV: levofloxacin, GEN: gentamicin; TRS: trimethoprim/sulfamethoxazole, TIG: tigecycline; R = resistant, I = intermediate, S = sensitive, n.d.: not determined
